# Daratumumab‐Based Combinational Therapy as Second‐Line Treatment of Relapsed‐Refractory Multiple Myeloma: A Single‐Center Experience

**DOI:** 10.1002/cnr2.70367

**Published:** 2025-11-29

**Authors:** Oscar C. Y. Yang, Yi‐Hao Chiang, Caleb Gonshen Chen, Yu‐Cheng Chang, Ming‐Chih Chang, Huan‐Chau Lin, Johnson Lin, Ken‐Hong Lim

**Affiliations:** ^1^ Department of Internal Medicine, Division of Hematology and Oncology MacKay Memorial Hospital Taipei Taiwan; ^2^ Department of Medical Research, Laboratory of Good Clinical Research Center MacKay Memorial Hospital New Taipei City Taiwan; ^3^ Department of Medicine MacKay Medical College New Taipei City Taiwan

**Keywords:** monoclonal antibodies, multiple myeloma, oncology, therapeutic uses

## Abstract

**Background:**

Daratumumab represents the first‐in‐class fully humanized monoclonal antibody that targets CD38 for the treatment of relapsed/refractory multiple myeloma (RRMM). Evidence from randomized controlled trials has shown daratumumab to be efficacious in the setting of second‐line combinational therapy for pretreated multiple myeloma. However, real‐world evidence that supports daratumumab use in daily clinical practice remains scarce.

**Aim:**

The primary objective of this study was to describe the real‐world clinical and adverse effects observed in RRMM patients receiving daratumumab as second‐line therapy.

**Methods:**

This was an observational case series with a retrospective chart review of pretreated multiple myeloma patients who received daratumumab‐based combinational therapy at an academic medical center. The primary end point was progression‐free survival. Additional end points included overall response rates, adverse effects of daratumumab therapy, and subsequent treatment options following daratumumab.

**Results:**

Seventeen patients were included. The overall response rate of daratumumab in our patients with RRMM was 13/17 (76.5%), and the median progression‐free survival was 20 months when daratumumab was used in the second‐line setting. Common adverse effects included neutropenia (52.9%), thrombocytopenia (64.7%), anemia (35.7%), and pneumonia (35.3%). On follow‐up, 10 patients remained alive at the experimental cut‐off date, with 2 patients kept on daratumumab‐based combinational therapy; 5 patients were switched to carfilzomib‐based therapy; and 3 received best supportive care.

**Conclusion:**

In our single‐center experience with Taiwanese RRMM patients, daratumumab in combinational therapy showed promising efficacy, and modest tolerance in the second‐line setting.

## 1 Introduction

Multiple myeloma (MM) accounts for approximately 17% of hematologic malignancies worldwide [1], with growing incidence in regions with aging populations [2]. The outcome of myeloma patients in Asian populations has steadily improved over the past decades due to the availability of generic systemic therapeutic agents including proteasome inhibitors (PIs) and immunomodulatory drugs (IMiDs), providing therapeutic options in both newly diagnosed and relapsed/refractory disease while mitigating financial constraints [3, 4].

Daratumumab is a fully human IgG1κ monoclonal antibody targeting CD38, a transmembrane glycoprotein highly expressed on malignant plasma cells [5]. Among CD38‐directed agents, daratumumab exhibits the highest complement‐dependent cytotoxicity (CDC) at low concentrations and binds a unique epitope, resulting in broad‐spectrum antimyeloma activity [6]. It also mediates antibody‐dependent cellular cytotoxicity (ADCC), phagocytosis (ADCP), and immunomodulatory effects [7]. Subcutaneous (SC) daratumumab, now approved alongside the intravenous (IV) formulation, demonstrates comparable efficacy, improved safety, and significantly shorter administration times—factors especially relevant in outpatient and resource‐constrained settings [8].

Supported by multiple trials, daratumumab‐based regimens are approved for use in both front‐line MM therapy and as a second‐line option in relapsed or refractory multiple myeloma (RRMM) [9, 10]. In the Phase 3 POLLUX trial, daratumumab plus lenalidomide and dexamethasone (DRd) significantly improved 12‐month progression‐free survival (PFS) (83.2% vs. 60.1%; HR 0.37, 95% CI 0.27–0.52, *p* < 0.001) compared to lenalidomide and dexamethasone alone [9]. At the 4‐year follow‐up, DRd was associated with a significantly higher overall response rate (ORR) (93% vs. 76%), including higher rates of very good partial response (PR) (81% vs. 49%) and complete response (CR) (57% vs. 24%; all *p* < 0.0001) [11]. Therefore, daratumumab has been shown to be efficacious as a second‐line therapeutic option for pretreated MM.

An important caveat to the available clinical evidence on daratumumab is the relative paucity of real‐world data that reinforces the efficacy and safety of second‐line daratumumab for RRMM in the Asian population [12, 13]. In the larger clinical trials for daratumumab, < 20% of the patients were of Asian descent [9]. Given that Asian patients tend to be more susceptible to the side effects of systemic therapy [14], the safety profile of daratumumab is of particular relevance to real‐world practice in East Asia. We herein document a single‐center experience of using daratumumab in Taiwan, in the context of DRd‐ or DVd‐based second‐line or later therapy for treating 17 RRMM patients at Mackay Memorial Hospital.

## 2 Materials and Methods

### 2.1 Study Design and Subjects

This was a single‐center, retrospective, longitudinal cohort study of MM patients aged 18 years and older. The study period spanned from May 2020 to August 2022. Seventeen patients were identified as having received daratumumab as second‐line or later therapy. Medical records were reviewed to collect clinical data, including demographics, treatment response, PFS, overall survival (OS), adverse events, and subsequent management.

Inclusion criteria were age ≥ 18 years, a confirmed diagnosis of MM per International Myeloma Working Group (IMWG) criteria, and receipt of daratumumab‐based combination therapy (DRd or DVd) as second‐line or later treatment during the study period.

Exclusion criteria included prior daratumumab exposure in the first‐line setting, age < 18 years, baseline alanine aminotransferase (ALT) or aspartate aminotransferase (AST) > 45 U/L, total bilirubin > 1.5 mg/dL, or known active hepatitis B or C virus infection (HBV/HCV).

**FIGURE 1:**
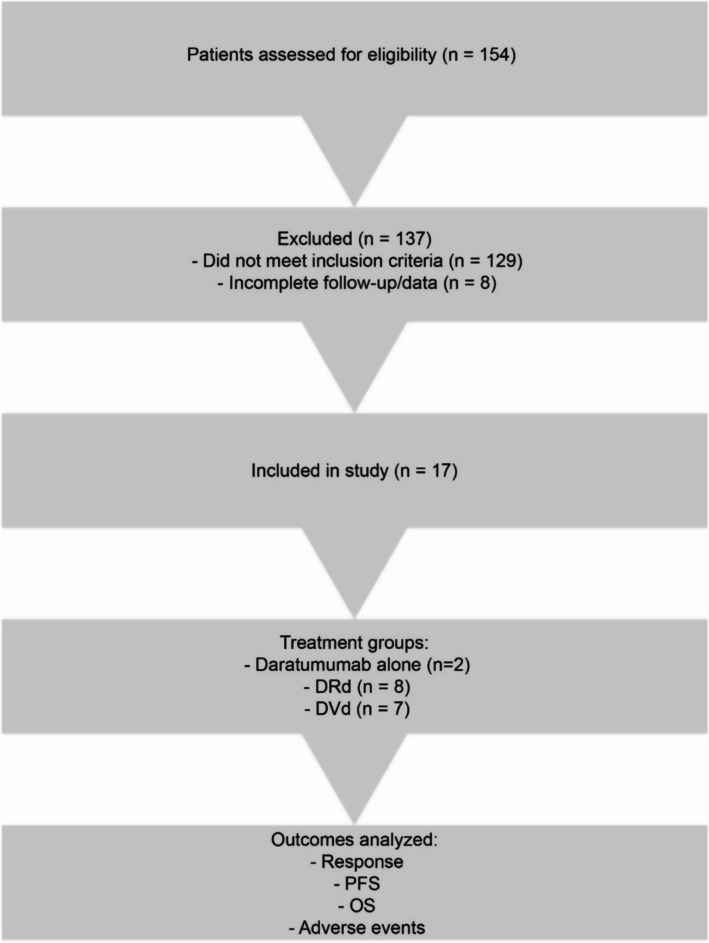
Flow diagram of patient selection and treatment allocation. DRd, daratumumab, lenalidomide, and dexamethasone; DVd, daratumumab, bortezomib, and dexamethasone; OS, overall survival; PFS, progression‐free survival.

A detailed overview of the patient screening and selection process is illustrated in Figure 1. The study protocol was approved by the institutional ethics review board of Mackay Memorial Hospital and conducted in accordance with the Declaration of Helsinki (approval code: 23MMHIS202e).

### 2.2 Daratumumab Treatment

Daratumumab was administered at a dose of 16 mg/kg in combination with either dexamethasone and lenalidomide (DRd) or dexamethasone and bortezomib (DVd).

For the DRd regimen, daratumumab was administered IV once weekly (Days 1, 8, 15, and 22) for 8 weeks (cycles 1–2), every 2 weeks (Days 1 and 15) for 16 weeks (cycles 3–6), and every 4 weeks thereafter [9].

For the DVD regimen, daratumumab was administered weekly (Days 1, 8, and 15) during cycles 1–3, every 3 weeks (Day 1) during cycles 4–8, and every 4 weeks thereafter [10].

Pre‐medications included methylprednisolone and diphenhydramine, administered 1 h prior to infusion.

### 2.3 Definitions and Statistical Analysis

Treatment response was evaluated according to the IMWG response criteria. The ORR was defined as the proportion of patients achieving CR, very good partial response (VGPR), or PR.

OS was defined as the time from the first daratumumab dose to death from any cause. PFS was defined as the time from the first daratumumab dose to documented disease progression or death. Patients who remained alive without progression were censored at their last follow‐up date.

Kaplan–Meier curves were used to estimate OS and PFS. Data were analyzed using GraphPad Prism version 9 (GraphPad Software, San Diego, CA, USA). A two‐tailed *p* < 0.05 was considered statistically significant.

## 3 Results

### 3.1 Patient Characteristics

The characteristics of the enrolled patients are listed in Table 1. From May 2020 to August 2022, 17 patients with RRMM who had received at least one prior line of therapy were treated with combinational pharmacotherapy inclusive of daratumumab. The median age of the patients was 68 years old and 52.9% were male. Four patients (23.5%) had complex cytogenetics. Two patients (11.8%) had an international staging system (ISS) score of one; four patients (23.5%) had an ISS score of two, and nine patients (52.9%) had an ISS score of three. In terms of myeloma classification, 10 (58.8%) patients were of the IgG subtype, five (29.4%) patients had IgA myeloma, and two (11.8%) had light chain myeloma. Ten (58.8%) patients expressed kappa light chain and seven (41.1%) expressed lambda light chain. For treatment, 12 patients (70.5%) had received 1 prior line of therapy, and five patients (29.4%) had received two or more lines of therapy prior to commencing the study. All of the 17 (100%) patients had received bortezomib; 16 (94%) received thalidomide; six (35.3%) received lenalidomide, and six (32.5%) received autologous stem cell transplantation (ASCT). Eight patients (47%) were treated with the DRd regimen that consisted of daratumumab, lenalidomide, and dexamethasone; seven patients (41%) received DVd—daratumumab, bortezomib, and two (11.7%) were treated with daratumumab alone. At our institution, 22 infusions of daratumumab were completed in six patients (35%), while 11 (65%) were not able to do so.

**TABLE 1:** Patient characteristics at time of daratumumab/combination therapy.

*N* = 17	
Prescription date interval	2020/5–2022/8
Age (median)	49–79 (68)
Gender—no. (%)	
Male	9 (52.9%)
Female	8 (47%)
Creatinine	
< 1.2	10
1.2–2.0	3
> 2.0	4
ISS	
1	2
2	4
3	9
Chromosome	
Normal	11
Complex	4
Prior lines of therapy (LoT)—no. (%)	
0, as in 1st LoT	0
1, as in 2nd LoT	12 (70.5%)
≥ 2, as in ≥ 3rd LoT	5 (29.4%)
Prior exposure—no. (%)	
ASCT	6 (32.5%)
Thalidomide	16 (94%)
Bortezomib	17 (100%)
Lenalidomide	6 (35.3%)
Daratumumab combination—no. (%)	
No, dara alone	2 (11.7%)
DRd	8 (47%)
DVd	7 (41%)
Complete 22 infusion—no. (%)	
Yes	6 (35%)
No	11 (65%)
Type	
IgG	10
IgA	5
Light chain	2
Light chain	
Kappa	10
Lambda	7

Abbreviations: ASCT, autologous stem cell transplant; DRd, daratumumab in combination with lenalidomide plus dexamethasone; DVd, daratumumab in combination with bortezomib plus dexamethasone; IgA, immunoglobulin A; IgG, immunoglobulin G; ISS, international staging system; LoT, line of therapy.

### 3.2 Daratumumab Efficacy

**FIGURE 2:**
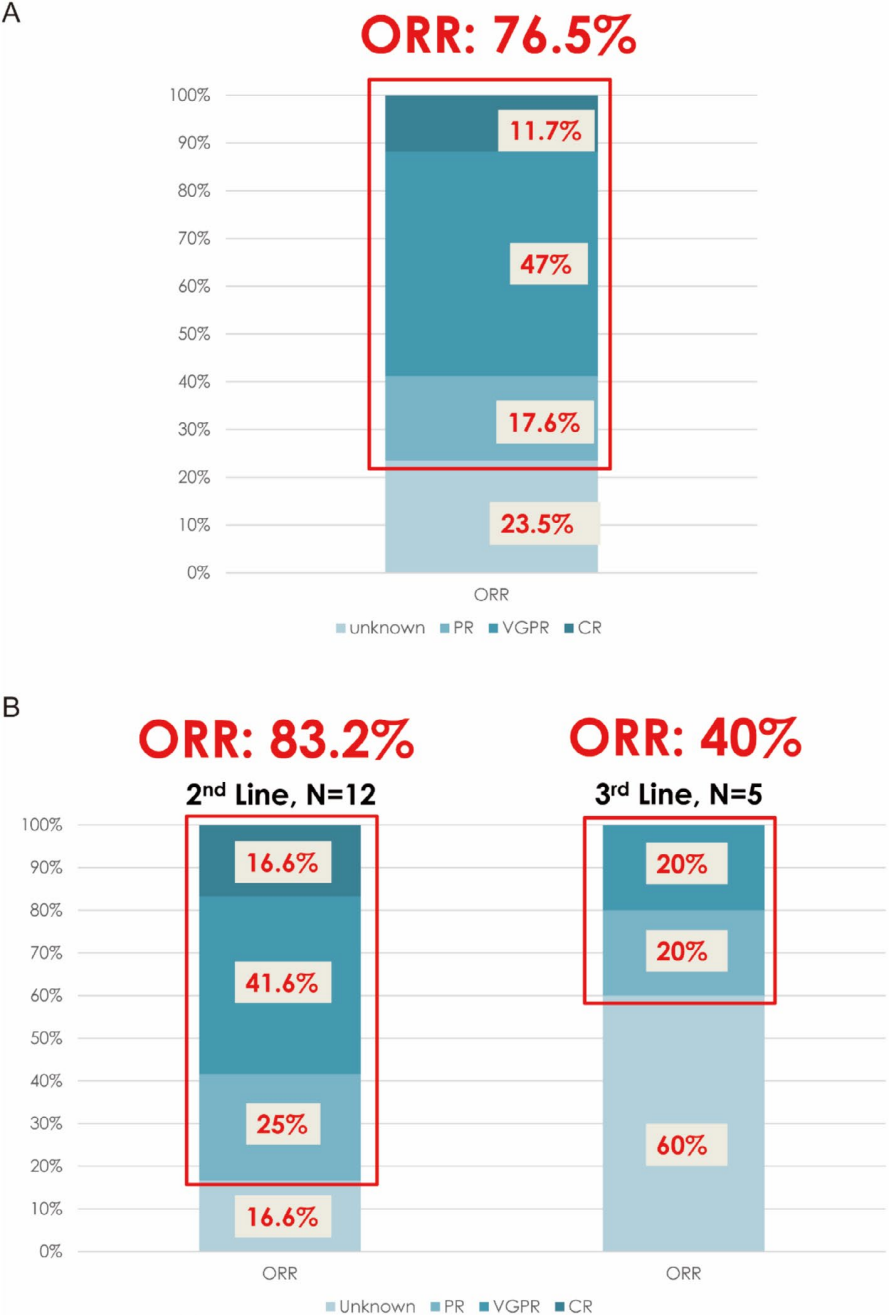
Treatment response to daratumumab/combination in patients with RRMM. (A) Overall response rate. (B) Response rate by line of treatment. CR, complete response; ORR, overall response rate; PR, partial response; VGPR, very good partial response.

**FIGURE 3:**
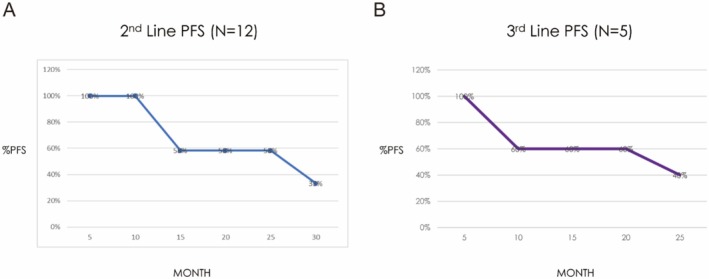
Progression‐free survival of daratumumab/combination in patients with RRMM—by LoT. (A) PFS for Dara/Combo as second LoT. (B) PFS for Dara/Combo as third LoT. PFS, progression‐free survival.

Six out of the 17 (35.3%) enrolled patients were able to receive the full 22 cycles of daratumumab. The ORR of daratumumab in combination therapy in patients with RRMM was 76.5%, with 11.7% achieving CR, 47% achieving VGPR, and 17.6% with PR (Figure 2A). When stratified by the number of previous lines of treatment, daratumumab in combination therapy as second‐line treatment (*N* = 12) achieved an ORR of 83.2% (CR 16.5%, VGPR 41.6%, and PR 25%) (Figure 2B), while daratumumab as third‐line treatment (*N* = 5) achieved an ORR of 40% (CR 20%, VGPR 20%) (Figure 2B). The median PFS when using daratumumab as second‐line treatment was 20 months (Figure 3A), and the median PFS when using daratumumab as third‐line treatment was 15 months (Figure 3B).

Exploratory subgroup analysis did not demonstrate any strong trends in response or PFS by age, sex, or prior therapy. Patients with ISS stage I disease (*n* = 2) appeared more likely to achieve VGPR or better, compared to those with Stage II or III (*n* = 15), though no formal statistical analysis was performed due to limited sample size.

### 3.3 Daratumumab Safety

Low‐grade hematological disorders were common at baseline prior to the administration of daratumumab, with six (35.3%) patients with documented anemia, 11 (64.7%) cases of thrombocytopenia, and nine (52.9%) patients with neutropenia, though ≥ grade three adverse events were rare (Table 2). Post‐daratumumab combinational therapy, there were eight (47.1%) cases of anemia, 12 (70.6%) cases of thrombocytopenia, and 11 (64.7%) cases of neutropenia, with an accompanying increase in ≥ grade three adverse events. Two patients developed low‐grade elevation in liver enzymes.

**TABLE 2:** Adverse events from daratumumab/combo therapy.

	Pretreatment (any grade)	Pretreatment (≥ Grade 3)	Posttreatment (any grade)	Posttreatment (≥ Grade 3)
IRR	0 (0.0%)	0 (0.0%)	4 (23.5%)	2 (11.7%)
Anemia	6 (35.3%)	1 (5.9%)	8 (47.1%)	3 (17.6%)
Thrombocytopenia	11 (64.7%)	1 (5.9%)	12 (70.6%)	3 (17.6%)
Neutropenia	9 (52.9%)	0 (0.0%)	11 (64.7%)	2 (11.7%)
Elevation of liver enzymes	0 (0.0%)	0 (0.0%)	2 (11.7%)	0 (0.0%)
Pneumonia	3 (17.6%)	0 (0.0%)	6 (35.3%)	1 (5.9%)
UTI	1 (5.9%)	0 (0.0%)	1 (5.9%)	0 (0.0%)
Bacteremia	1 (5.9%)	0 (0.0%)	3 (17.6%)	2 (11.7%)

Abbreviations: IRR, infusion‐related reaction; UTI, urinary tract infection.

Infusion‐related reactions (IRRs) occurred in four patients (23.5%), including two cases (11.7%) that reached Grade 3 Severity. No patient required treatment discontinuation due to IRRs.

Infectious complications were notable, with six cases (35.3%) of pneumonia, including one fatal case of pneumocystis jirovecii pneumonia (PJP) with neutropenic sepsis. There was one low‐grade urinary tract infection and three cases of ≥ Grade 3 bacteremia, due to *Escherichia coli* , *MRSA*, and *PJP*, respectively.

### 3.4 Subsequent Management After Daratumumab

At the time of data cut‐off, five patients had deceased, two were lost to follow‐up, and 10 remained alive (Table 3). One patient underwent ASCT following daratumumab‐based therapy, having achieved disease stabilization with a PFS of 15 months (Table 3). Of the five patients who had expired, one passed away due to PJP infection; one died from MRSA‐related grade four neutropenic sepsis, another patient expired from intracranial hemorrhage with cardiorespiratory compromise while on daratumumab—for this particular patient, the platelet count post‐daratumumab was 87 000/μL; and two others passed away after disease progression and switching to carfilzomib‐based therapy. For the remaining 10 patients who remained alive, subsequent treatment regimens are shown in Table 3. Two patients were able to be kept on daratumumab‐based combinational therapy, five patients were switched to carfilzomib‐based therapy, and three received best supportive care.

**TABLE 3:** Subsequent management following daratumumab/combo therapy.

Patient	Subsequent treatment	Current status	Dara PFS (months)	Overall survival (months)
1	ASCT	Lost to F/U	15	NR
2	Kept on 2L DVd	Alive	10	10
3	Kd → Ikd	Alive	1	20
4	BSC	Expired	1	2
5	BSC	Alive	1	NR
6	KRd	Alive	14	27
7	KRd	Alive	15	29
8	BSC	Expired	8	9
9	BSC	Expired	11	11
10	BSC	Alive	12	12
11	K	Alive	9	28
12	K	Expired	6	15
13	Kept on 3L DVd	Alive	25	25
14	Lost to F/U	Lost to F/U	3	NR
15	Kd → Ikd	Expired	11	20
16	BSC	Alive	10	33
17	Kd	Alive	9	16

Abbreviations: 2L, second‐line; 3L, third line; ASCT, autologous stem cell transplant; BSC, best supportive care; DVd, daratumumab in combination with bortezomib plus dexamethasone; F/U, follow‐up; Ikd, isatuximab in combination with carfilzomib plus dexamethasone; K, carfilzomib monotherapy; Kd, carfilzomib in combination with dexamethasone; KRd, carfilzomib in combination with lenalidomide plus dexamethasone; NR, not recorded; PFS, progression‐free survival.

## 4 Discussion

In this retrospective single‐center study, we evaluated the real‐world use of daratumumab‐based combination therapy in 17 Taiwanese patients with RRMM. We observed an ORR of 76.5%, with 58.7% achieving VGPR or better, and a median PFS of 20 months in the second‐line setting. Daratumumab was generally well tolerated, although infective complications were relatively common, including one fatal case of PJP. One patient proceeded to ASCT following daratumumab therapy. While DRd and DVd are established regimens in the Western setting, their real‐world application in East Asia—where daratumumab is not uniformly available as frontline therapy—remains clinically relevant. In our center, daratumumab is commonly introduced in the second‐line setting due to local reimbursement policies and treatment accessibility.

Certain clinical characteristics in our cohort were comparable to those in the POLLUX and CASTOR trials, including patient age and measurable disease burden [9, 10]. However, our population had a greater proportion of patients with ISS stage III disease (52.9% vs. 20.1% in POLLUX and 23.5% in CASTOR) and lower previous ASCT exposure (32.5% vs. ~63% in trials), reflecting real‐world treatment limitations and later‐stage disease [9, 10]. Prior exposure to IMiDs was also more frequent in our population. A subanalysis of the POLLUX trial by Suzuki et al. reported similar efficacy and safety of DRd in East Asian patients compared to the broader trial population [15]. Our findings are broadly consistent with Suzuki et al., although that analysis was conducted in a clinical trial setting.

Despite real‐world differences in demographic and disease characteristics, DRd and DVd regimens in the current study demonstrated encouraging activity in the second‐line setting, with an ORR of 83.2%—comparable to outcomes seen in the clinical trials (POLLUX trial: ORR for the experimental arm was 92.9% [9]; CASTOR trial: ORR was 82.9% [10]). In contrast, 5 of 17 patients receiving daratumumab in the third‐line setting achieved an ORR of 40.0%, aligning with other real‐world studies where later‐line daratumumab regimens yielded lower ORRs (24.4%–56.3%) [16, 17]. This supports the concept that daratumumab may be more effective when used earlier in the treatment course, consistent with broader clinical practice guidelines.

No unexpected safety signals were observed, and the adverse event profile was consistent with previous reports, with hematologic toxicity and infections being the most common events [12, 18]. However, the rate of Grade ≥ 3 neutropenic infections was notable. One patient developed fatal PJP, and another experienced MRSA‐related sepsis. Prophylactic antibiotics or antifungal agents, including trimethoprim‐sulfamethoxazole for PJP, were not routinely administered during the study period. Similarly, the use of granulocyte‐colony stimulating factor (G‐CSF) was not standardized, which may have influenced infection outcomes.

Beyond infections, other aspects of safety were also consistent with prior reports. IRRs occurred in 23.5% of patients in our cohort, including two Grade 3 events, all of which were managed without treatment discontinuation. This IRR rate is consistent with those reported in the pivotal trials, where rates ranged from 26% to 48% depending on the infusion route and regimen [9, 10]. The absence of IRR‐related discontinuations supports the tolerability of daratumumab in this East Asian cohort.

Although not observed in our cohort, cardiac toxicity and poor stem cell mobilization have been described in the Asian context [19, 20]. Though rare, Jandial et al. reported a case of sudden cardiac arrest following daratumumab infusion in an Indian patient, raising the question of underlying susceptibility in East Asian populations [19]. Mishra et al. have also described poor stem cell mobilization after daratumumab‐based therapy in newly diagnosed patients, which may have implications for transplant planning [20]. Further investigation is warranted to explore ethnicity‐linked pharmacodynamics and toxicity profiles of daratumumab‐based regimens.

In terms of subsequent therapies post‐daratumumab, our practice favored carfilzomib‐based combinations (Table 3), whereas other cohorts have reported the use of bendamustine, pomalidomide, or cytotoxic regimens such as DCEP [12]. The heterogeneity in third‐line approaches reflects real‐world flexibility and the influence of local treatment availability and reimbursement policies. Further comparative data will be important to optimize sequencing after daratumumab exposure.

This study has several limitations. The small sample size limited the ability to perform subgroup analyses, precluded formal statistical comparisons, and reduced generalizability. Over half of the cohort did not complete the full 22‐course daratumumab regimen. The follow‐up duration was modest, and the absence of standardized prophylaxis—such as PJP prevention or consistent use of G‐CSF—may have influenced infection outcomes. In addition, our analysis was limited to DRd and DVd regimens, without evaluation of more recently adopted combinations such as DKd [21] or DPd [22], which may reflect local prescribing patterns and access constraints. Nonetheless, real‐world data from East Asian populations remain scarce, and our findings provide preliminary insight into the tolerability and sequencing of daratumumab in this underrepresented group. These results reinforce the utility of daratumumab in RRMM when used in early relapse and underscore the value of region‐specific real‐world studies.

## 5 Conclusion

In summary, our single‐center experience in Taiwan demonstrated that daratumumab‐based combination therapy is active and generally well tolerated in patients with RRMM receiving second‐line or later treatment. Infectious complications were not uncommon, and patterns of ASCT use and infection prophylaxis in our cohort differed from those reported in clinical trials. These findings contribute to the growing body of real‐world evidence supporting daratumumab's role in RRMM management and underscore the importance of region‐specific data in guiding clinical decision‐making.

## Author Contributions

Design of study: patient enrollment and data collection: Yi‐Hao Chiang, Caleb Gonshen Chen, Yu‐Cheng Chang, Ming‐Chih Chang, Huan‐Chau Lin, Johnson Lin, Ken‐Hong Lim. Data analysis: Yi‐Hao Chiang and Oscar Yang. Manuscript writing: Oscar C.Y. Yang. Manuscript revision: Yi‐Hao Chiang, Oscar C.Y. Yang, Caleb Gonshen Chen, Yu‐Cheng Chang, Ming‐Chih Chang, Huan‐Chau Lin, Johnson Lin, Ken‐Hong Lim.

## Ethics Statement

The study protocol was approved by the institutional ethics review board of Mackay Memorial Hospital and complied with the Declaration of Helsinki (approval code: 23MMHIS202e).

## Consent

Patient consent to the study was carried out according to the approved study protocol (approval code: 23MMHIS202e).

## Conflicts of Interest

The authors declare no conflicts of interest.

## Data Availability

The data that support the findings of this study are available from the corresponding author upon reasonable request.
